# Bio-implant as a novel restoration for tooth loss

**DOI:** 10.1038/s41598-017-07819-z

**Published:** 2017-08-07

**Authors:** Dong-Joon Lee, Jong-Min Lee, Eun-Jung Kim, Takashi Takata, Yoshihiro Abiko, Teruo Okano, David W. Green, Masaki Shimono, Han-Sung Jung

**Affiliations:** 1 Division in Anatomy and Developmental Biology, Department of Oral Biology, Oral Science Research Center, BK21 PLUS Project, Yonsei University College of Dentistry, Seoul, Korea; 2 Department of Oral Pathology, Faculty of Dentistry, Hiroshima University, Hiroshima, Japan; 3 Division of Oral Medicine and Pathology, Department of Human Biology and Pathophysiology, School of Dentistry, Health Sciences University of Hokkaido, Hokkaido, Japan; 4 Institute of Advanced Biomedical Engineering and Science, Tokyo Women’s Medical University, Tokyo, Japan; 5 Department of Pathology, Tokyo Dental College, Chiba, Japan; 6 Applied Oral Science, Faculty of Dentistry, The University of Hong Kong, Hong Kong, SAR China

## Abstract

A dental implant is used to replace a missing tooth. Fixing the implant in its natural position requires the engineering of a substantial amount of conformal bone growth inside the implant socket, osseointegration. However, this conventional implant attachment does not include the periodontal ligament (PDL), which has a fundamental role in cushioning high mechanical loads. As a result, tooth implants have a shorter lifetime than the natural tooth and have a high chance of infections. We have engineered a “bio-implant” that provides a living PDL connection for titanium implants. The bio-implant consists of a hydroxyapatite coated titanium screw, ensheathed in cell sheets made from immortalized human periodontal cells. Bio-implants were transplanted into the upper first molar region of a tooth-extraction mouse model. Within 8 weeks the bio-implant generated fibrous connective tissue, a localised blood vessel network and new bone growth fused into the alveolar bone socket. The study presents a bio-implant engineered with human cells, specialised for the root connection, and resulted in the partial reconstruction of a naturalised tooth attachment complex (periodontium), consisting of all the principal tissue types, cementum, PDL and alveolar bone.

## Introduction

Dental implants are the most improved treatment for teeth that have been either extracted or have been ejected as a result of periodontal disease. Although implant treatment has a high success rate, there are some fundamental vulnerabilities associated with osseointegration, the healing mechanism between the bone and the titanium implant fixture^1, 2^. The osseointegration process at the implant surface, however, does not incorporate the periodontal ligament (PDL) space. Natural teeth are connected to surrounding fibrous tissues for efficient biological function performance^3^.

Specifically, The PDL is the fibrous connective tissue between the tooth-root and the alveolar bone. The PDL fibres play essential roles in the absorption of occluding forces. The PDL is also a prominent region for proprioception, which contributes to the collective function among the teeth, masticatory muscles and the temporomandibular joint, all under the control of the central nervous system^4, 5^.

Since dental implants are secured only by osseointegration, they are more susceptible to infections. This arises because without the existence of periodontal sensory mechanisms, including proprioception, pain perception is absent when periodontal diseases, including peri-implantitis arise and undermining shape bone resorption occurs. In one study to address this, a bio-hybrid implant was devised. Embryonic dental follicle tissue encircled onto the implant, bio-hybrid implant, comprised of whole periodontal tissue formation surrounding the dental implant tissue, was developed^3^. Moreover, a similar bio-hybrid implant trial, incorporated living bone tissue as a substitute for cementum, was reported^6^.

We report the use of cell sheets, derived from immortalized human cells, instead of embryonic tissue for implantation and to regenerate a living periodontium. The cell sheet technique was developed to avoid the limitations of tissue reconstruction using biodegradable scaffolds or single cell suspension injections^7^. This technique has been popular. Several cell sheets have been employed therapeutically, for cornea, bladder, and heart regeneration^8^. Cell sheets have also facilitated periodontal tissue regeneration in previous studies^9–11^. Accordingly, human PDL cells were directed in to a cell sheet in osteogenic differentiation medium and then transplanted on periodontal fenestration defects in immunodeficient rats. The transplanted PDL cell sheets induced periodontal regeneration with a cementum layer and Sharpey’s fibres^9^. A tri-layered PDL cell sheet derived from canine PDL tissue was transplanted into artificial 3-wall infrabony defects with complete cementum removal in canine models, and periodontal regeneration was observed^10^. Another study reported that cell sheet fragments of canine PDL stem cells and platelet-rich fibrin granules enhanced periodontal healing in avulsed tooth re-implantation in a canine model^11^. Knowing that cell sheets alone can integrate and regenerate compound periodontal tissues we explored the idea of marrying them to the base of dental implants to generate a proper biological attachment.

The dental implant used in the present study was designed and fabricate for implementation in a small mouse models. The thread of the titanium implant was coated with an ultrathin layer of hydroxyapatite (HA) to enhance the bone fusion process. The HA-coated implant had primarily been used in clinical dental implant treatments, as the bone healing process around the implant is enhanced by the hydroxyapatite layer^12^. The HA-coated implant used in the present study simulated human dental implant treatment.

Human periodontal cells, including cementoblasts and PDL cells, were used in the present study. The human cementoblasts were obtained from healthy premolar extracts for orthodontic treatment^13^, and the human PDL-like cell line was derived from human cementifying fibroma (HCF) cells^14^. Both cell lines were immortalized by transfection with the telomerase catalytic subunit, the hTERT gene. These cell lines are referred to as immortalized human periodontal ligament cells (ihPDLs) and immortalized human cementoblasts (ihCEMs). We also employed green fluorescence protein (GFP)-tagged human umbilical vein endothelial cells (HUVECs) to induce blood supply and accelerate periodontium regeneration. Previous studies have shown that HUVECs promote vasculogenesis through co-culture with tumour tissues *in vitro* ^15^. In addition, among clinical dental implant treatments, the blood supply is essential for bone healing and periodontium survival^16, 17^. Moreover, epithelial cell rests of Malassez (ERM) were used to promote periodontium regeneration. The ERM is able to regulate the width of the PDL space, induce the nerve ending growth, and induce cementoblast differentiation^18^. The ERM cell line used in the present study was porcine derived^19^.

The four cell types, contributing to periodontium regeneration were applied as single- or multi-layered cell sheets. The transplanted cell sheets which encircled the fixture were comprised of only a single cell type or a combination of various cell types. Eight weeks after transplantation, the transplanted cell sheets formed PDL-like connective tissue or plate-shaped calcified tissue on the surface of the implant fixture. They also induced alveolar bone remodelling. In the present study, we demonstrated that immortalized human cells, HUVECs and ERM cells could be sustained *in situ*, and engineer an attachment between the fixture and alveolar bone. These cells form fibrous and calcified tissues in the expected regions.

These results demonstrate the potential of regenerating periodontal tissue using compound cell sheets around the dental implant without sacrificing the embryo. In addition, this study presents the principles of the cell types required to regenerate periodontal tissue around an implant.

## Results

### Implant preparation, transplantation procedure and immortalized human cell characteristics

**Figure 1:**
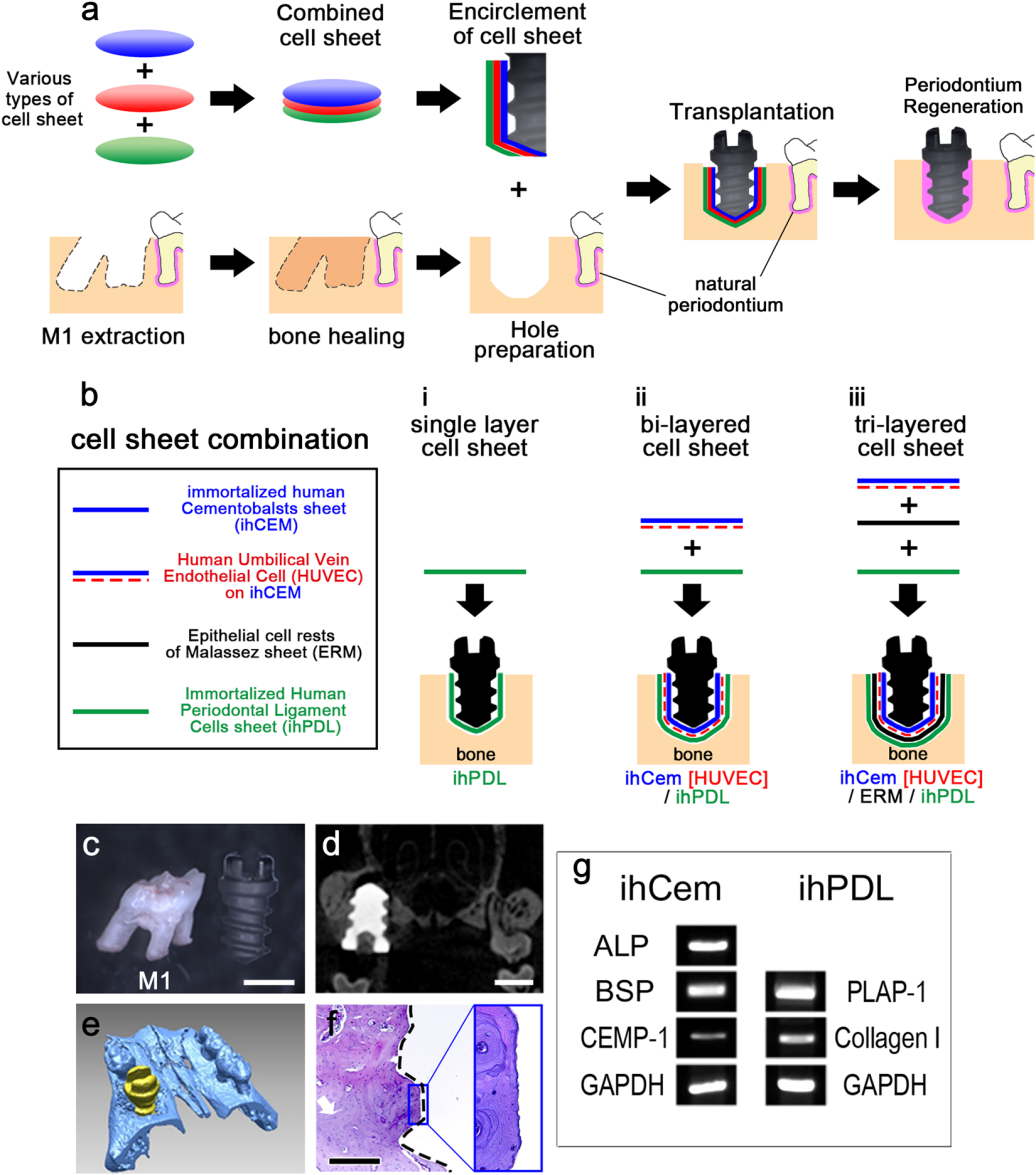
Transplantation of a bio-implant in a tooth extraction model and characteristics of the immortalized human cells. (**a**) Schematic representation of the bio-implant procedure. The main purpose of this study is periodontium regeneration around implant fixture. (**b**) Combination of cell sheets used in this study. Various types of cell sheet (panel a) are described. (**c**) Maxillary first molar and HA-coated dental implant fixture. (**d**) Micro CT image of 8 weeks after transplantation of the HA-coated fixture shows the alveolar bone compactly healed around the fixture. (**e**) 3D reconstruction of the micro CT results at 8 weeks after transplantation of the HA-coated fixture. (**f**) HE-stained image at 8 weeks after transplantation of the HA-coated fixture showing osseointegration between the alveolar bone and the fixture. Black dashed line shows the margin of the removed implant fixture. (**g**) RT-PCR results of immortalized human cells (cropped gel image). The ihCEMs expressed ALP, BSP and CEMP-1. The ihPDLs expressed PLAP-1 and ColI. Full-length gel is presented in Supplementary Figure 6. Scale bars, (**b**,**c**) 1 mm; (**e**) 200 μm.

To implant a fixture into maxillary alveolar bone, the right first molar was extracted from 3-week-old BALB/c nude mice. The opposite side of the first molar was maintained for mastication, and allowed to heal for 6 weeks (Fig. 1a). After the alveolar bone healed, the fixture was wrapped with a cell sheet and implanted into a tooth-extracted region of a nude mouse. The cell sheet comprised various combinations of cell types. For investigating the functions of the cell sheet combinations, 10 nude mice were used for each combination. Figure 1b illustrates how various types of cell sheets were used in each case. In each case of single layer, bi-layered and tri-layered cell sheets﻿, the ihPDLs sheet, the HUVECs on ihCEMs sheet, and the ERMs sheet were used as component(s) of cell sheet combination or not.

The HA-coated dental implants were designed to be small enough to endure the force of grit blasting prior to HA coating and subsequent manipulation in the oral space of mice (Fig. 1c). Similar to dental implants for clinical use, the experimental dental implants had threads and a driver slot on the head. These implants resembled the conditions for human dental implant treatment.

Scanning electron microscopy (SEM) images of the surfaces of dental implants were generated. Although the titanium dental implant before HA coating had a smooth surface, the HA-coated dental implants had rough surfaces and enlarged surface areas (Supplementary Fig. 1a,b). These features are completely analogous to human HA-coated dental implants^20, 21^. The HA thickness was 1.86 μm [±0.16 μm standard deviation (SD)] on average (measured in 4 positions of 7 randomly selected samples, data not shown).

In addition, an HA-coated fixture was implanted in the right maxillary first molar extracted region without a cell sheet, acting as a positive control (Supplementary Fig. 1c). The alveolar bone was healed and positioned between fixture threads at 8 weeks after implantation. Micro-computed tomography (CT) images (Fig. 1d) and 3D reconstructions (Fig. 1e) showed compacted alveolar bone healing around the dental implant and a properly positioned fixture. Haematoxylin and eosin (HE) staining after sectioning showed osseointegration between the alveolar bone and the fixture (Fig. 1f).

To validate the functional characteristics of the ihCEMs and ihPDLs used in this study, we examined mRNA expression of the mineralization-related genes, cementoblast and PDL markers (Fig. 1g and Supplementary Fig. 6). The ihCEMs showed expression of mineralization-related genes, including alkaline phosphatase (ALP), bone sialoprotein (BSP), and cementoblast marker cementum protein 1 (CEMP-1, also known as CP-23). The ihPDLs expressed collagen type I (Col I) and periodontal-ligament-associated protein-1 (PLAP-1), which is known as a PDL specific marker^22^.

### Periodontium-like tissue formation between the implant fixture and alveolar bone

**Figure 2:**
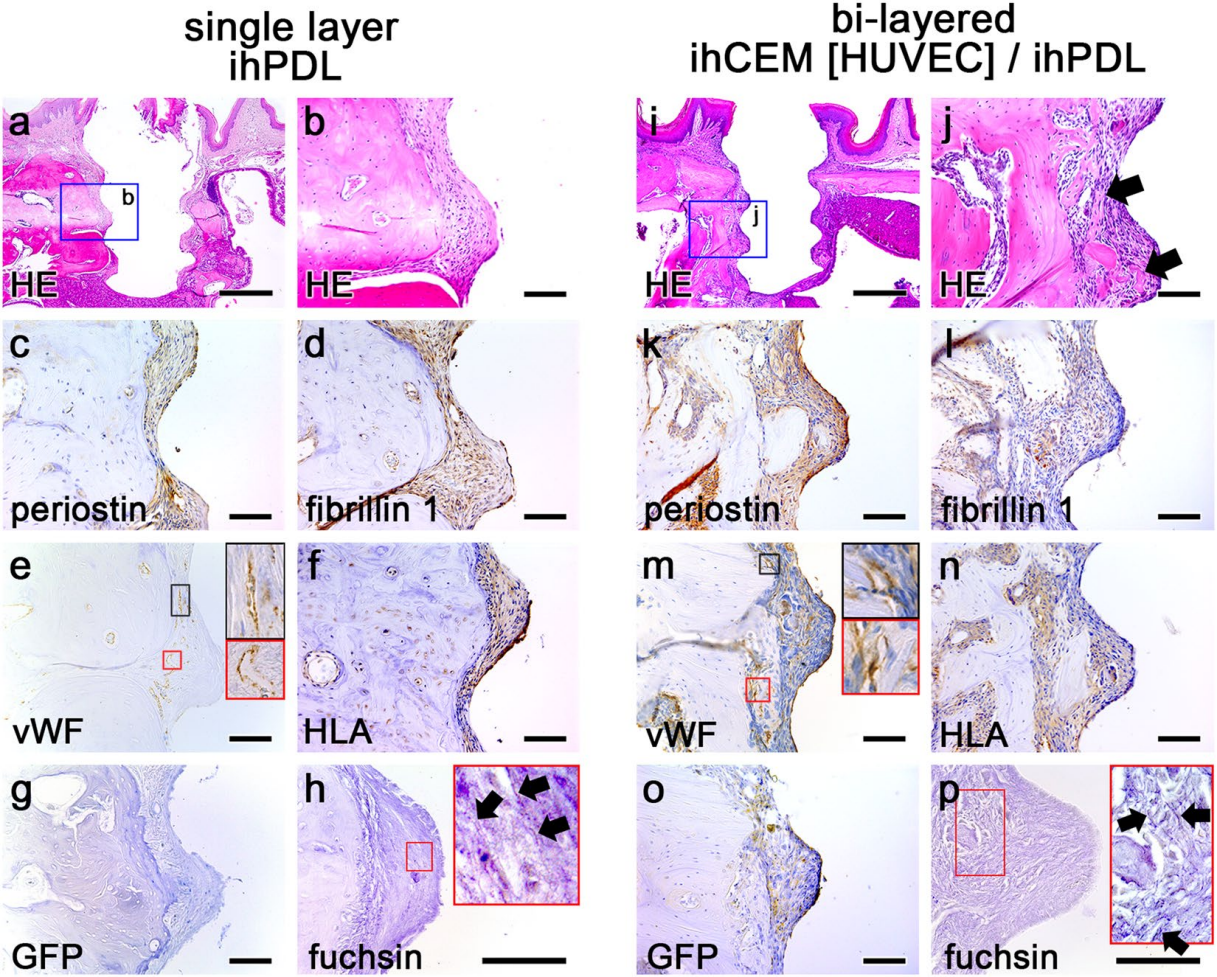
Periodontium-like tissue formation by transplanted cell sheets. (**a**) HE-stained image at 8 weeks after ihPDLs sheet transplantation. PDL-like connective tissue is observed between the fixture and the alveolar bone. (**b**) High magnification of panel a. The alignment of the PDL-like tissue is parallel to the fixture. (**c**,**d**) IHC of the PDL markers, periostin and fibrillin 1. The periostin and fibrillin 1 staining was positive around the PDL-like tissue. (**e**) IHC of the blood vessel marker, vWF. Blood vessels were observed in the alveolar bone and PDL-like tissue. (**f**) IHC of HLA. The PDL-like tissue showed a strong HLA signal, and the signal was also observed in some areas of the alveolar bone. (**g**) IHC of GFP. In the ihPDLs sheet transplantation, GFP was not detected. (**h**) Resorcin-fuchsin staining. Arrows show the oxytalan fibres in the PDL-like connective tissue. (**i**) HE-stained image at 8 weeks after ihCEM [HUVEC]/ihPDL bi-layered cell sheet transplantation. (**j**) High magnification of panel g. The arrows indicate remodelled bone. The alignment of the fibrous tissue is perpendicular to the fixture. (**k**,**l**) IHC of periostin and fibrillin 1. Positive periostin and fibrillin 1 signalling were detected in the PDL-like tissue. (**m**) IHC of vWF. Blood vessels were observed around the remodelled bone and in the PDL-like tissue. (**n**) IHC of HLA. The PDL-like tissue shows positive HLA signalling. (**o**) IHC of GFP. This shows blood vessels in the PDL-like tissue originated from the GFP tagged HUVECs. (**p**) Resorcin-fuchsin staining. Arrows indicate the oxytalan fibres in the PDL-like tissue. Scale bars, (**a**,**i**) 500 μm; (**b**–**h**,**j**–**p**) 100 μm.

To investigate whether the transplantation of dental implants covered by cells could regenerate *in situ* periodontal tissue, ihPDLs were used as a component of the cell sheet (Fig. 1b,i and Supplementary Fig. 1d). At 8 weeks after transplantation, the implant encircled with an ihPDLs sheet formed PDL-like tissue between the alveolar bone and the implant fixture (Fig. 2a). The alignment of PDL tissue was vertical and parallel to the fixture (Fig. 2b). To confirm whether this newly formed connective tissue was PDL, immunohistochemistry (IHC) was performed using the well-known two PDL markers, periostin and fibrillin 1 (Fig. 2c,d). In addition, engaged blood vessels were observed in the newly formed PDL-like tissue as confirmed by positive expression of von Willebrand factor (vWF, an endothelial cell marker) (Fig. 2e). Positive human leukocyte antigen (HLA) staining by IHC confirmed that the PDL-like tissue originated from human PDL cells (Fig. 2f). Indeed, the PDL-like connective tissue originated from human cells. IHC of GFP was performed to compare this single layer ihPDLs sheet transplantation with other cell sheet combinations including GFP-tagged HUVECs. GFP was not detected in this ihPDLs sheet (in absence of HUVECs) transplanted sample (Fig. 2g). Moreover, through resorcin-fuchsin staining with oxone treatment, oxytalan fibres were detected in the PDL-like connective tissue (Fig. 2h arrows).

On the contrary, single layer of ihCEMs sheet transplantation with implant showed normal osseointegration occurred 8 weeks after transplantation (Supplementary Fig. 2a,b). In Masson’s trichrome (M-T) staining, blue-stained fully calcified tissue was observed in alveolar bone, close to the fixture (Supplementary Fig. 2c). In addition, HLA-positive ihCEMs were observed in alveolar bone close to the fixture (Supplementary Fig. 2d boxes). However, osteocytes far from the fixture did not themselves express HLA, suggesting they were not derived from the ihCEMs.

### The contribution of HUVECs and ihCEMs to the multi-layered cell sheet

To regenerate periodontal tissue, HUVECs were incorporated into the cell sheet. The HUVECs were cultured for 7 days onto previously formed ihCEMs or ihPDLs sheet, respectively. The HUVECs were seeded onto both cell sheets at a seeding density of 50,000 HUVECs/cm^2^. The HUVECs formed a circular vasculature shape on the cell sheet after culturing with the endothelial growth medium (EGM) for 7 days. These sheets were named ‘ihCEM [HUVEC]’ and ‘ihPDL [HUVEC]’ (Supplementary Fig. 3). Although the HUVECs were differentiated on the ihPDL sheet, the ihPDL [HUVEC] was contracted after it was detached from the temperature-responsive culture dish (data not shown). This appearance was not observed with the ihCEM [HUVEC].

The HUVECs were wrapped in direct contact with the alveolar bone and the ihCEMs or ihPDLs faced the fixture (Supplementary Fig. 4a). At 8 weeks after transplantation, the HUVECs on the ihPDLs sheet (ihPDL [HUVEC]) showed large circular vessels in the alveolar bone close to the fixture (Supplementary Fig. 4b). The vessel formation was confirmed by IHC using vWF (Supplementary Fig. 4c). Soft tissue was not observed between the healed alveolar bone and the fixture. Despite the PDL-like tissue, the alveolar bone tightly filled the space between the fixture and the implant socket wall. The HUVECs on the ihCEMs sheet (ihCEM [HUVEC]) formed irregularly shaped vessels near the fixture (Supplementary Fig. 4d,f) and newly formed calcified tissue was observed on both sides of the vessels (Supplementary Fig. 4e arrows). Newly formed cementum-like calcified tissue was confirmed by M-T staining. In the M-T staining, the blue colour indicates hard tissue and the red spots in the blue hard tissue indicate immature calcifying tissue. Some areas of the plate-shaped calcified tissue were stained red.

To determine whether the combination of ihCEMs and ihPDLs could simultaneously produce PDL and cementum, an ihPDLs sheet was layered onto an ihCEM [HUVEC] sheet (ihCEM [HUVEC]/ihPDL) and transplanted alongside the fixture (Fig. 1b,ii). The ihCEM [HUVEC] sheet was bi-layered with the ihPDLs sheet in a manner in which the HUVECs faced the ihPDLs sheet. When this bi-layered cell sheet was used to wrap the fixture, the ihCEMs made contact with the fixture and the ihPDLs were positioned on the outside. At 8 weeks after transplantation, PDL-like fibrous tissue formed and remodelled bone was observed growing into the space between the fixture threads (Fig. 2j arrows). In some areas of the fibrous connective tissue, the fibre direction improved their function (perpendicular to the fixture). Periostin and fibrillin 1 were observed in the PDL-like tissue, including in the functional alignment region (Fig. 2k,l). The fibrillin 1 signal was particularly strong on the surface facing the fixture. The detected vWF signal was strong in the areas surrounding the remodelling bone (Fig. 2m). To confirm whether the connective tissue between the alveolar bone and the fixture was PDL originated from human cells, IHC against HLA was performed (Fig. 2n). An HLA-positive signal was observed in the fibrous tissues around the fixture. GFP was also detected around the blood vessels in the PDL-like tissue (Fig. 2o), and we observed that PDL-like connective tissue formed by the bi-layered cell sheet contained oxytalan fibres (Fig. 2p arrows). However, the directions of the oxytalan fibres were irregular and their lengths were shorter than that of natural PDL (Supplementary Fig. 5c).

### Addition of ERMs in cell sheets induced calcification

**Figure 3:**
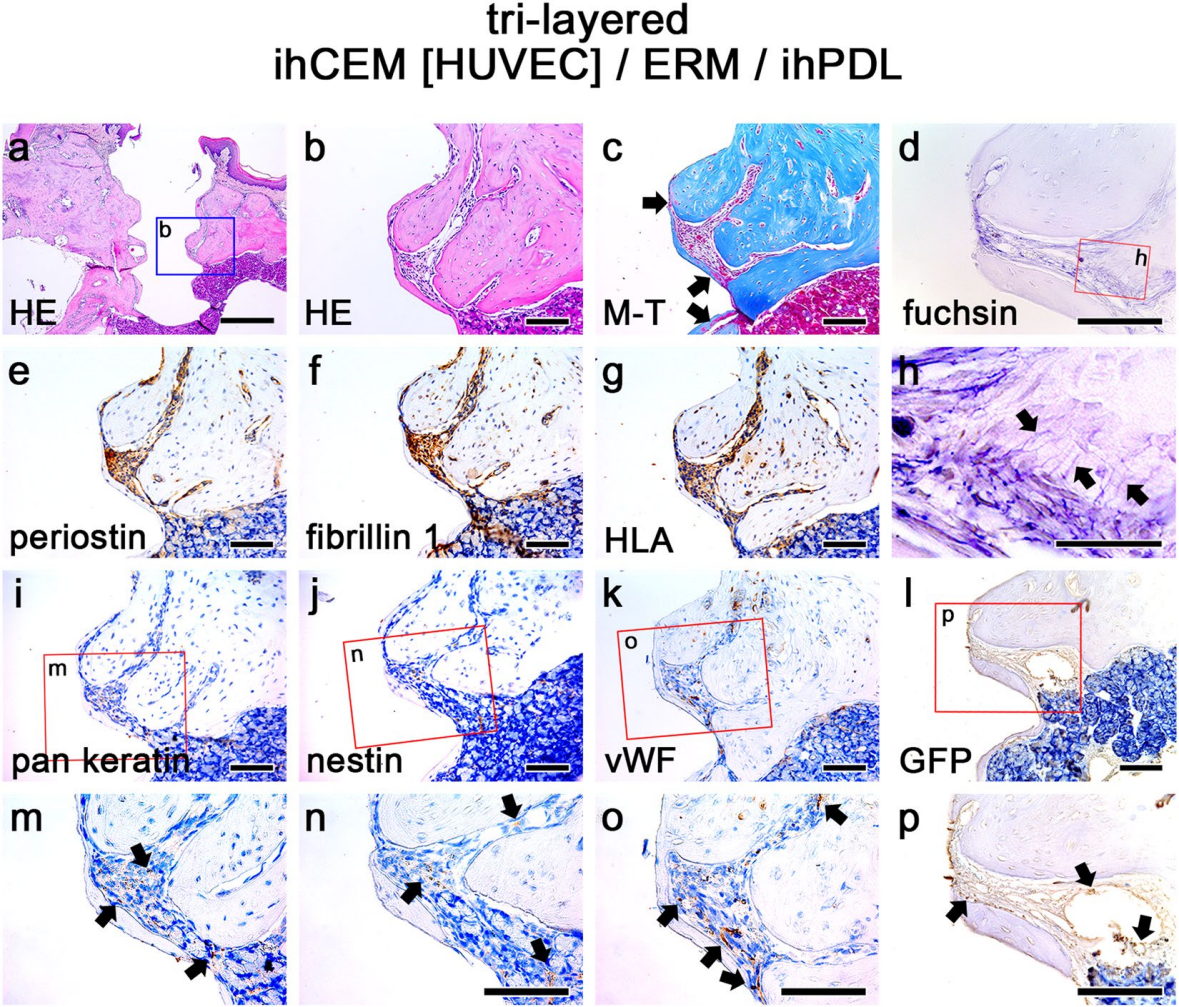
ERM cells play roles in PDL width regulation and calcification. (**a**) HE-stained image at 8 weeks after ihCEM [HUVEC]/ERM/ihPDL tri-layered cell sheet transplantation. (**b**) High magnification of panel a. Thin, calcified tissue formed on the surface of the fixture. The alveolar bone healed well in the direction of the fixture. The PDL-like tissue formed between the thin, calcified tissue and the alveolar bone. (**c**) M-T-stained image. The arrows indicate the red areas on the blue calcified tissue, indicating newly formed calcifying tissue. (**d**,**h**) Resorcin-fuchsin staining. Arrows indicate the oxytalan fibres in the PDL-like tissue. (**e**,**f**) IHC of periostin and fibrillin 1. In the PDL-like connective tissue, both PDL markers were strongly positive. (**g**) IHC of HLA. All PDL-like tissues and the areas of alveolar bone near the fixture showed positive signalling. (**i**,**m**) IHC of pan keratin (an ERM marker). Pan keratin positive cells were observed in the PDL-like tissue (arrows). (**j**,**n**) IHC of nestin (a nerve marker). Nestin positive cells were observed in the PDL-like tissue (arrows). (**k**,**o**) IHC of vWF. Small blood vessels were formed in the PDL-like tissue and alveolar bone (arrows). (**l**,**p**) IHC of GFP. Arrows shows blood vessels in the PDL-like tissue originated from GFP tagged HUVECs. Scale bars, (**a**) 500 μm; (**b**–**g**,**i**–**p**) 100 μm; (**h**) 50 μm.

To more closely resemble the cell composition present in natural tooth periodontium, ERMs were incorporated into tri-layered cell sheet (ihCEM [HUVEC]/ERM/ihPDL) around implants and subsequently transplanted (Fig. 1b,iii). For the tri-layered sheet, plate-shaped calcified tissue was observed on the surface of the fixture (Fig. 3a,b). Connective tissue was formed between this calcified tissue and the alveolar bone. The width of the connective tissue between the fixture and the alveolar bone was less than that formed by the transplanted bi-layered (ihCEM [HUVEC]/ihPDL) sheet. The alveolar bone facing the newly formed connective tissue healed faster than that of the transplanted bi-layered sheet, where bone remodelling was not detected a﻿t all. According to M-T staining, the plate-shaped calcified tissue was newly formed (Fig. 3c). The arrows in Fig. 3c indicate red staining on blue calcified tissue, indicating newly formed (immature) calcified tissue. Resorcin-fuchsin staining showed oxytalan fibres in the connective tissue between the alveolar bone and plate-shaped calcified tissue (Fig. 3d,h). The direction of the oxytalan fibres appeared to originate from the plate-shape calcified tissue and ran perpendicular to the PDL fibres (Fig. 3h arrows). Periostin and fibrillin 1 staining were observed in the PDL-like tissue (Fig. 3e,f). HLA was detected in the PDL-like tissue, and some alveolar bone osteocytes close to the fixture were also positive for HLA (Fig. 3g). To confirm the remaining ERMs, IHC against pan keratin was performed (Fig. 3i,m). Pan keratin-positive cells were observed in the PDL-like tissue (Fig. 3m arrows). IHC against nestin was also performed to detect the presence of nerve tissue. Nestin-positive cells were observed in the PDL-like tissue (Fig. 3j,n arrows).Blood vessels were engaged in the PDL-like tissue (Fig. 3k,o arrows) with vWF, and the GFP signals were detected around the blood vessels in the connective tissue (Fig. 3l,p).

## Discussion

It has been previously reported that dental implants covered in embryonic dental follicle cell sheets, termed a bio-hybrid implant, led to synthesis of complete periodontal tissue^3^. However, embryonic cells are clinically risky due to their tumorigenic potential and ethical issues arising from the sacrifice of embryos. To bridge ethical problems, we developed a new type of bio-implant coated with cells unsourced from embryonic tissues. Instead we assembled constitutive human adult, human umbilical cord and adult porcine derived cells as alternatives to embryonic cells. Dental implants were HA-coated, resembling human dental implants, but designed for transplantation into mice. The fixtures were manufactured as small as possible to endure grit blasting prior to HA coating, and the fixture had a driver slot on the head to provide conditions similar to human dental implant treatment.

Cell sheets were manufactured and manually wrapped around the screw thread fixtures﻿. Regardless of assembled cell types the cell sheet covered fixture generated sufficient tissue to heal the wound. Specific anatomical characteristics emerged depending on the types of cells present inside the sheets. For instance, ihPDLs produced PDL-like connective tissue around the dental implant fixture following transplantation, whereas the ihCEMs with ERMs formed calcified tissue. However, we could not confirm whether the calcified tissue was cementum, due to the absence of a cementum specific marker. Although we used two types of antibodies known as cementum markers, cementum attachment protein (CAP) and CEMP1, both antibodies showed false-positive signals in the broad connective tissue area around the fixture transplantation (data not shown).

**Figure 4:**
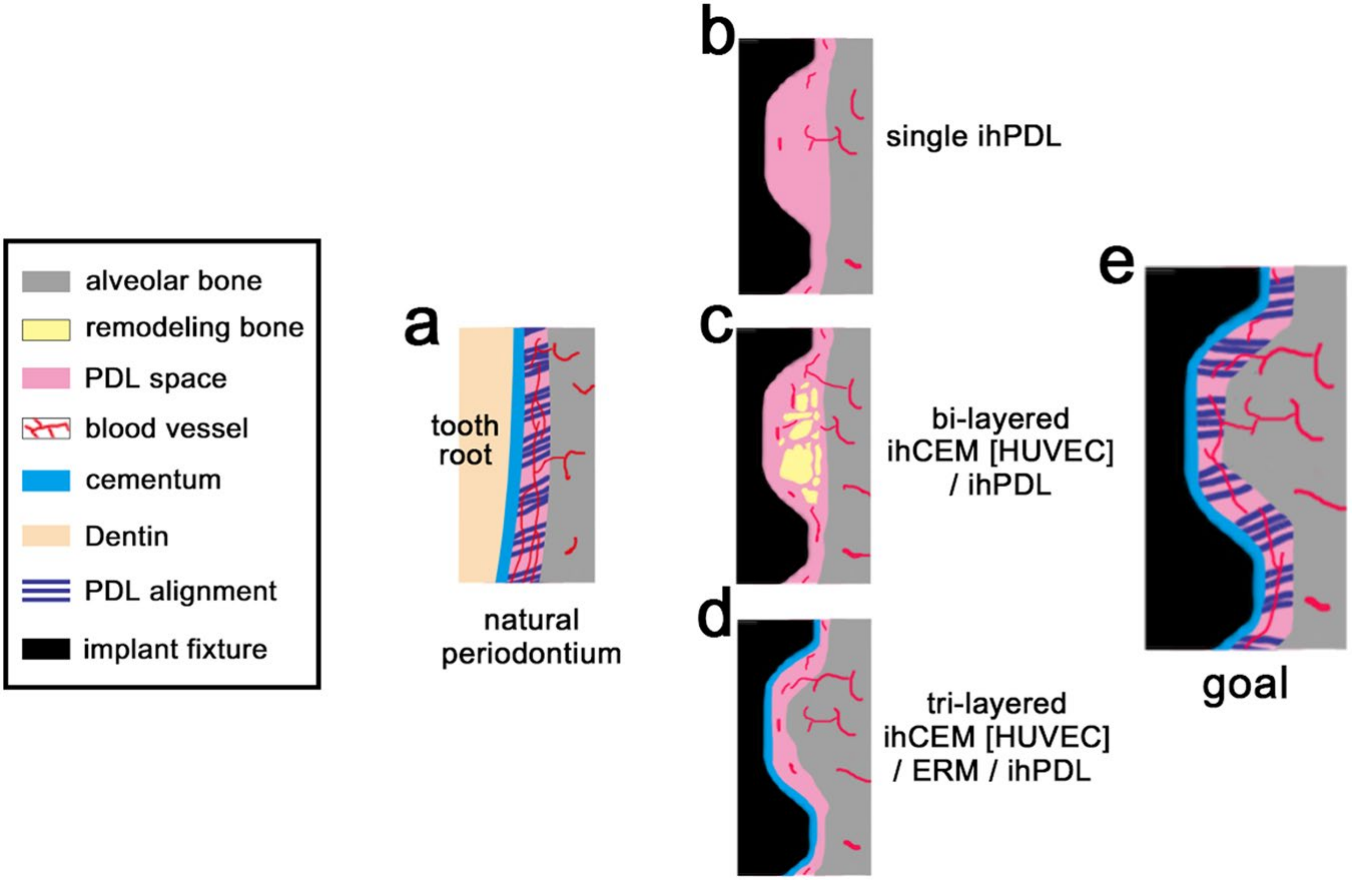
Schematic representation of the bio-implant. (**a**) The natural periodontium contains cementum, PDL connecting alveolar bone and cementum, and blood vessels. (**b**) The ihPDLs sheet bio-implant shows PDL-like connective tissue formation; however, the vertically aligned PDL-like tissue cannot function. A few blood vessels are engaged in the PDL-like tissue. (**c**) The ihCEM [HUVEC]/ihPDL bi-layered cell sheet bio-implant. Bone remodelling occurred between the threads of the fixture and the blood vessels around the remodelled bone. (**d**) The ihCEM [HUVEC]/ERM/ihPDL tri-layered cell sheet bio-implant shows rapid bone healing and thin cementum-like calcified tissue on the fixture. The PDL space was narrower than the bi-layered bio-implant. (**e**) Final goal of the bio-implant. The PDL connects alveolar bone with properly formed cementum on the fixture. The width of the PDL space is precisely regulated for optimal functioning.

Among implants covered with ihPDLs sheets, a layer of PDL tissue developed with vertical alignment running parallel to the fixture (Fig. 2b). Function was presumably lacking, because the PDL did not in any way innervate into the fixture due to the absence of a proper cementum layer (Fig. 4b).

The transplanted ihCEMs sheet could not form calcified tissue, but they survived and remained in the alveolar bone in the vicinity of the fixture (Supplementary Fig. 2d). Since alveolar osteocytes near the fixture do not express HLA, it can be inferred that they are not differentiated from ihCEMs and it is not the cementum-like tissue.

For clinical treatment of dental implants to succeed in creating viable, living tissue grafts and host integration, a good blood supply is necessary^16, 17^. Absence of such a blood supply system will inevitably lead to tissue necrosis and ejection of the implant from the bone socket. Therefore, it was imperative that we employed blood vessel forming endothelial cells (HUVECs) into the tissue layers that would have the *in vitro* potential to connect to the host blood system. Prior to transplantation, HUVECs were cultured in layers onto ihCEMs and ihPDLs sheets for 7 days, respectively. The protocol for layering HUVECs over cell sheets made from CEMs and PDLs had been previously developed^23^. In this type of assembly ihPDLs sheet and HUVECs showed cell sheet shrinkage, whereas, ihCEMs sheet with HUVECs did not. In addition, we observed that only the ihPDL cells could proliferate with the EGM. Moreover, excessive cell proliferation in the ihPDLs sheet caused inordinate tension between the cells. Hence, in the bi-layered and tri-layered cell sheet experiments, we used the ihCEM [HUVEC] sheets only, and not the ihPDL [HUVEC] sheets.

At 8 weeks after transplantation of the ihCEM [HUVEC] and ihPDL [HUVEC] sheet fixtures, large vessels formed close to the fixture. This excessive vasculogenesis reflected the direction in which the cell sheet was wrapped. Specifically, HUVECs localised themselves onto the alveolar socket wall and developed blood vessels, leading to rapid bone growth filling the space between the fixture and the alveolar bone. In this zone connective tissue was totally absent. Thus, to ensure periodontal tissue formation with connective tissue HUVECs should not be in contact with the socket wall, and that a high number of transplanted HUVECs w﻿as not necessary. Thus, the number of HUVECs spread onto the ihCEMs sheet was reduced from 50,000 to 10,000 cells/cm^2^ in subsequent experiments (Figs 2i–p and 3).

For the formation of cementum that the PDL can embed into, ihCEMs were added to the cell sheet combination. In the transplanted bi-layered cell sheet (ihCEM [HUVEC]/ihPDL), bone remodelling was detected in the space between the fixture threads, which filled the space along the contour of the fixture (Fig. 4c). In the remodelled bone, osteoblasts and osteoclasts were observed^24^. Some parts of the fibrous portion in the PDL-like tissue were horizontally aligned (perpendicular to the direction of fixture implantation). This alignment was similar to the Sharpey’s fibre in the natural PDL space. However, the embedding of PDL fibre into the cementum-like tissue was not detected; thus, this tissue could not be considered intact Sharpey’s fibres.

The tri-layered cell sheet (ihCEM [HUVEC]/ERM/ihPDL) produced newly formed calcified tissue on the surface of the fixture. This calcified tissue formed a thin plate-shape and functioned as cementum (Fig. 4d). It has been suggested that ERMs influence the ihCEMs layer to inducing calcification and mineralisation. As the result of rapid bone healing compared with the bi-layered (ihCEM [HUVEC]/ihPDL) sheet transplanted sample, it is inferred that ERMs significantly influenced bone remodelling. Considering that the regulation of the PDL width and the differentiation of cementoblasts are the known functions of the ERM, the results obtained for the tri-layered sheet are consistent with that.

The GFP expression in both the bi-layered and tri-layered cell sheet transplantation experiments proved that the transplanted HUVECs survived and engrafted well. Resorcin-fuchsin staining with oxone treatment showed the existence of oxytalan fibres. The oxytalan fibres are a normal component of the PDL in human and various animal species^25^. Oxytalan fibres run parallel to the tooth surface and bend to attach to cementum (Supplementary Fig. 5c arrowheads)^26^. The existence of oxytalan fibres in periodontal tissue regeneration have also been detailed as powerful evidence of intact PDL regeneration^27^. The detection of oxytalan fibres in our multi-layer tissue composite means that the transplanted ihPDL cells could form genuine PDL tissue. Although the direction and the length of oxytalan fibres were different from that in the natural PDL tissue, this phenomenon is thought to arise from the lack of intact cementum.

In conclusion, the present study demonstrates that immortalized human cementoblasts, immortalized human PDL cells, HUVECs and ERM cells play roles in fixture transplantation and alveolar bone remodelling in the socket wall. This study represents the potential of regenerating periodontium-like tissue around the dental implant without sacrificing embryos. However, the technique used in this study is inapplicable for human therapy since some used cells were immortalized and derived from a fibroma. In tests so far, human primary cells did not proliferate well and therefore could not been used in this study.

New technologies are needed to ensure that patient derived cells are combined into therapeutic sheets with exacting clinical functions and compound roles that lead to natural periodontium formation. Furthermore, the response of periodontal tissue sheets to masticatory forces, including hardness of feeding material must be properly validated before clinical application.

The objective of the study was to highlight, in proof-of-concept, the feasibility of combining the different human periodontal cells of the periodontium onto a titanium implant. These results are an indispensable first-step towards the clinical goal of culture a full thickness periodontium from compound cell sheets.

## Methods

All experiments were approved by Yonsei University College of Dentistry, Intramural Animal Use and Care Committee and they were performed in accordance with the guidelines of this committee.

### Animals

Female nude (nu/nu BALB/c/Bkl) mice (Narabiotech Co., Pyeongtaek, Korea) were housed in a temperature-controlled room (22 °C) under artificial illumination with a 12-hour light/dark cycle and 55% relative humidity. The mice were provided access to food and water ad libitum. All operational procedures were performed under deep anaesthesia.

### RNA preparation and reverse transcription-polymerase chain reaction analysis (RT-PCR)

Total RNA was isolated from confluent cell cultures using Trizol according to the manufacturer’s instructions. For cDNA synthesis, reverse transcription was performed using Maxime^TM^ RT PreMix (25081, Intron Biotechnology, Kor﻿e﻿a﻿﻿). And PCR was performed using the Thermal Cycler Dice TP600 (Takara, Japan) with AccuPower® PCR PreMix (K-2016, Bioneer, Korea). The amplification programme consisted of 40 cycles of the following: denaturation at 95 °C for 20 seconds, annealing at 57 °C for 20 seconds and extension at 72 °C for 70 seconds. The oligonucleotide RT-PCR primers for ColI, ALP, BSP, CEMP-1, PLAP-1 and glyceraldehyde-3-phosphate (GAPDH) are as follows:

**Table:** 

ColI	F 5′-ctgaccttcctgcgcctgatgtcc-3′	R 5′-gtctggggcaccaacgtccaaggg-3′
ALP	F 5′-aagtactggcgagaccaagc-3′	R 5′-agagggccacgaaggggaact-3′
BSP	F 5′-gaaccacttccccacctttt-3′	R 5′-tctgaccatcatagccatcg-3′
CEMP-1	F 5′-atgggcacatcaagcactga-3′	R 5′-ccccattagtgtcatcctgc-3′
PLAP-1	F 5′-tcccaaccaacattccattt-3′	R 5′-tcatctttggcactgttgga-3′
GAPDH	F 5′-tccaccaccctgttgctgta-3′	R 5′-accacagtccatgccatcac-3′

### Cell sheet culture

The ihPDLs and ERMs were cultured on conventional cell culture dishes with Dulbecco’s minimum Eagle’s medium (DMEM, 11995–065, Gibco, Life Technologies, USA) with 10% foetal bovine serum (FBS, 12484–020, Gibco, Life Technologies, USA), and 1% penicillin/streptomycin solution (P/S, Pen Strep, 15140-122, Gibco, Life Technologies, USA) under 37 °C, 5% CO_2_ conditions. The ihCEMs were cultured in Minimum essential medium alpha (MEM-α, 12571–063, Gibco, Life Technologies, USA) with 10% FBS and 1% P/S solution under 37 °C, 5% CO_2_ conditions. At 90% confluency in conventional dishes, the cells were detached using trypsin reagent (TrypLE, 12604–013, Gibco, Life Technologies, USA) and then harvested and spread onto temperature-responsive dishes (Nunc UpCell 3.5-cm dish, NUN-174904, Thermo Fisher Scientific, USA) at 50,000 cells/cm^2^. The cells typically reached 100% confluency on the temperature-responsive dishes after 3 days, and the media was changed using the same pre-warmed media. Subsequently, the cells were cultured for 2 additional days to generate an intact cell sheet. The dishes were incubated at 20 °C, 5% CO_2_ condition for 30 minutes to harvest the cell sheet. The cell sheets were layered via a simple pipetting method described in a previous report^28^.

### HUVEC cultures on cell sheets

GFP-expressing HUVECs were purchased from Angio-Proteomie (cAP-0001GFP,﻿ Boston, USA) and cultured in endothelial basal medium (EBM, cAP-03, Angio-Proteomie, USA) or endothelial growth media (EGM, cAP-02 Angio-Proteomie, USA) following the distributor’s instruction. The cell medium was changed every 2 days. After harvesting the HUVECs using trypsin, the cells were spread onto ihCEMs or ihPDLs sheets at 50,000 cells/cm^2^ in EGM. After culturing the HUVECs on the cell sheets for 7 days, the cell sheets were detached by changing the temperature to 20 °C, according to the manufacturer’s instructions.

### Transplantation of the bio-implant

The implants were generated from pure titanium and shaved to produce threads at the apical side, with a driver slot on the head. The thread of the implants was 1.42 mm in length and 1.00 mm in diameter. To promote cementum deposition around the implants, the surfaces were coated with HA at an average thickness of 1.86 *μ*m (Daechang metal., Co., Korea). The cell sheet was cut to the proper size (approximately 3 mm in diameter). The HA implant was positioned on the centre of the cut cell sheet vertically, and the cell sheet was wrapped around the HA implant by lifting up using a round-tipped tungsten needle.

The upper first molars of 3-week-old Balb/c nude mice were extracted under deep anaesthesia and permitted to heal for 6 weeks. Under deep anaesthesia, a pilot hole was prepared using a portable low speed engine with a 0.75-mm drill tip designed for these HA implants. Subsequently, the bio-implant was transplanted into the hole using a driver. The implant-transplanted mice were housed for 8 weeks in the animal room, described previously, for healing. The mice were subsequently euthanized with CO_2_.

### Immunohistochemistry (IHC)

The tissues were excised and immersed in 4% paraformaldehyde (PFA). After fixation, the tissues were decalcified in 10% sodium citrate and 22.5% formic acid for 6 weeks at 4 °C. Staining was performed on 6 μm paraffin-embedded sections. After deparaffinization, the slides were incubated with Proteinase K (10 μg/mL, AM2546, Thermo Scientific, USA) for 20 minutes at 37 °C or, for GFP, with pepsin (Digest-All™ 00–3009, Invitrogen, USA) for 10 minutes at 37 °C. Subsequently, the slides were incubated with antibodies against periostin (1:1,000 diluted, ab14041, Abcam plc, UK), fibrillin1 (1:500 diluted, ab53067, Abcam plc, UK), vWF (1:100 diluted, AB7356, EMD Millipore Co., USA), HLA (1:100 diluted, ab70328, Abcam plc, UK), nestin (1:200 diluted, MAB353, Millipore co. USA), panCK (1:50 diluted, MS-343-P0, Thermo Scientific, USA), or GFP (1:500 diluted, 2955 S, Cell Signalling Technology, USA) at 4 °C overnight. The specimens were sequentially incubated with secondary antibodies and streptavidin peroxidase. The results were visualized following staining with a diaminobenzidine (DAB) reagent kit (Invitrogen, USA). The sections were counterstained with Mayer’s haematoxylin. All specimens were observed using a stereomicroscope (MD5500D; Leica, camera: DFC495; Leica, Lens: HCX PL APO 409; Leica).

### Resorcin-fuchsin staining

Staining was performed on 6-μm paraffin-embedded sections, which was previously described. The slides were oxidized with Oxone® (diluted with distilled water to 10% m/v, 228036, Sigma-Aldrich, Switzerland) or distilled water for 10 minutes at room temperature. After they were rinsed with three distilled water washes (2 minutes each time), the slides were stained with resorcin-fuchsin solution (26370, Electron Microscopy Sciences, USA) for 10 minutes. They were differentiated in pure ethanol (3 changes with 10 immersions each time). After rinsing with running water, they were dehydrated and mounted. The sections were observed using the stereomicroscope mentioned previously.

## Electronic supplementary material

Supplementary information
