# Supplementary material for: Bio-implant as a novel restoration for tooth loss
Source: Sci Rep. 2017 Aug 7;7:7414. doi: 10.1038/s41598-017-07819-z (PMC5547161; doi:10.1038/s41598-017-07819-z)
Supplement: Supplementary file 1 — Supplementary information [file 41598_2017_7819_MOESM1_ESM.pdf]

Supplementary information

Bio-implant as a novel restoration for tooth loss

Dong-Joon Lee[1, †], Jong-Min Lee[1, †], Eun-Jung Kim[1], Takashi Takata[2], Yoshihiro Abiko[3], Teruo Okano[4], David W. Green[1], Masaki Shimono[5], Han-Sung Jung[1, 6, *]

[1]Division in Anatomy and Developmental Biology, Department of Oral Biology, Oral Science Research Center, BK21 PLUS Project, Yonsei University College of Dentistry, Seoul, Korea

[2]Department of Oral Pathology, Faculty of Dentistry, Hiroshima University, Hiroshima, Japan

[3]Division of Oral Medicine and Pathology, Department of Human Biology and Pathophysiology, School of Dentistry, Health Sciences University of Hokkaido, Hokkaido, Japan

[4]Institute of Advanced Biomedical Engineering and Science, Tokyo Women's Medical University, Tokyo, Japan

[5]Department of Pathology, Tokyo Dental College, Chiba, Japan

[6]Oral Biosciences, Faculty of Dentistry, The University of Hong Kong, Hong Kong, SAR

† These authors contributed equally to this work.

* To whom correspondence may be addressed: Han-Sung Jung, PhD, #601 Division in Anatomy and Developmental Biology, Department of Oral Biology, Yonsei University College of Dentistry, Seoul, 03722, Korea. Tel.: +82-2-2228-3064, Fax: +82-2-312-8012, E-mail: hsj8076@gmail.com

Supplementary information

This PDF file includes;

Supplementary Figure 1, 2, 3, 4, 5and 6

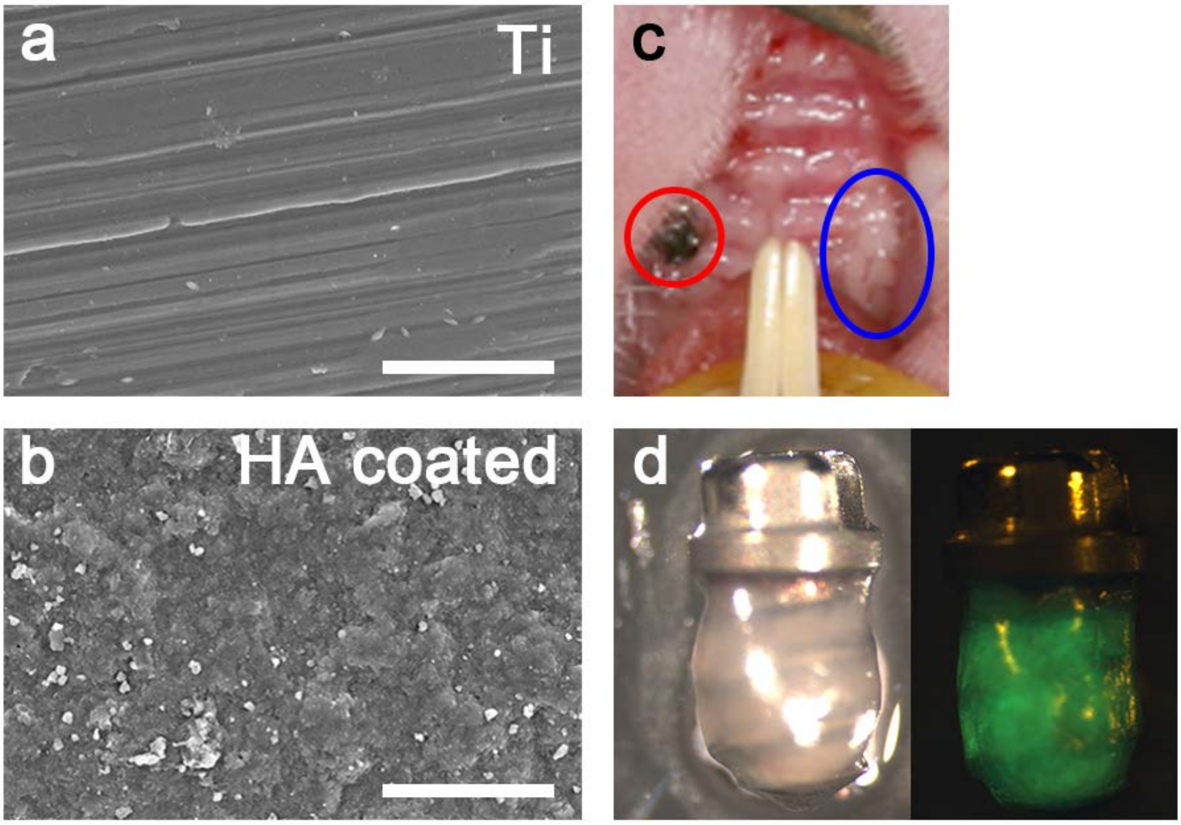

Supplementary Figure 1 | Implant fixture with a cell sheet wrapping

(a) Scanning electron microscopy (SEM) image of a titanium dental implant fixture without HA coating. (b) SEM image of a titanium dental implant fixture with HA coating. (c) Photograph of an HA-coated fixture transplanted in the right maxillary first molar region (red circle) and a natural first molar on the opposite side (blue ellipse). (d) Stereoscopic image of a cell sheet (PDL) wrapped the fixture. The cell sheet was stained with the Qtracker® cell labelling kit. Scale bars, 20 μm

Supplementary Figure 1. DJ. Lee *et al.*

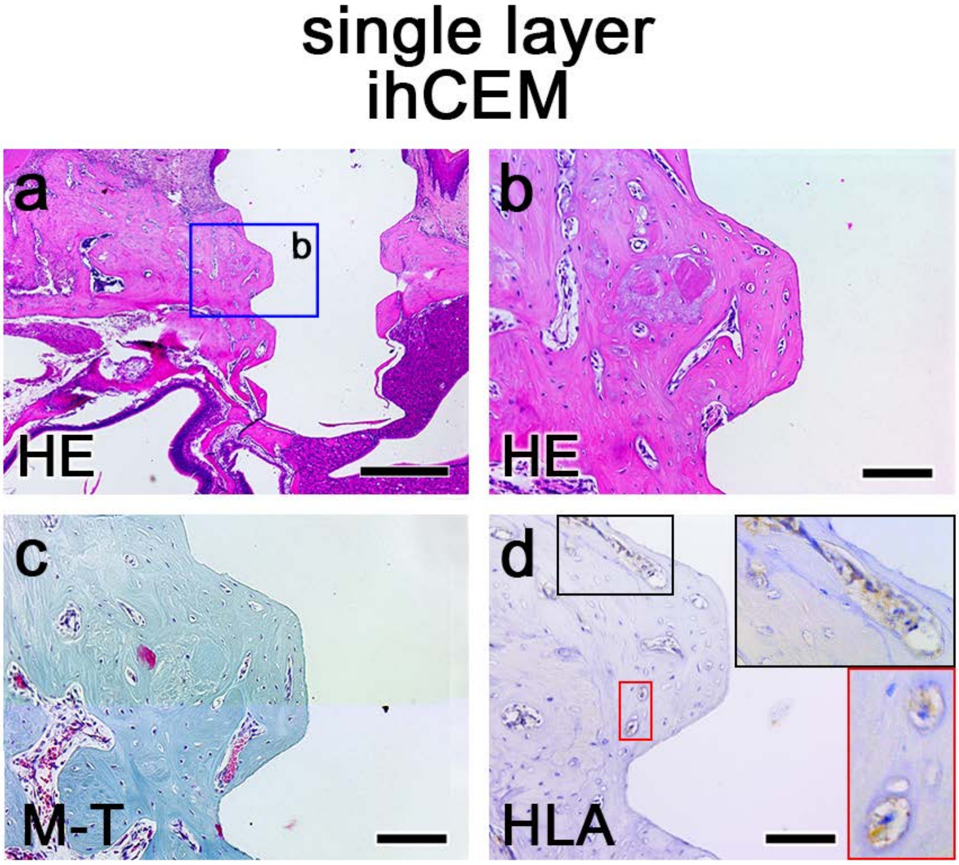

Supplementary Figure 2 | ihCEMs sheet formed normal osseointegration.

(a) HE-stained image at 8 weeks after ihCEMs cell sheet transplantation. (b) High magnification of panel a. Osseointegration is observed between the alveolar bone and the fixture. The soft tissue is not shown. (c) M-T stained image. Fully calcified blue stained tissue is observed around the fixture. (d) IHC of HLA. HLA positive ihCEMs were observed in the aloveolar bone of vicinity of the fixture. Alveolar osteocytes around the HLA positive ihCEMs do not express HLA. Scale bars, a, 500 μm ; b-d, 100 μm.

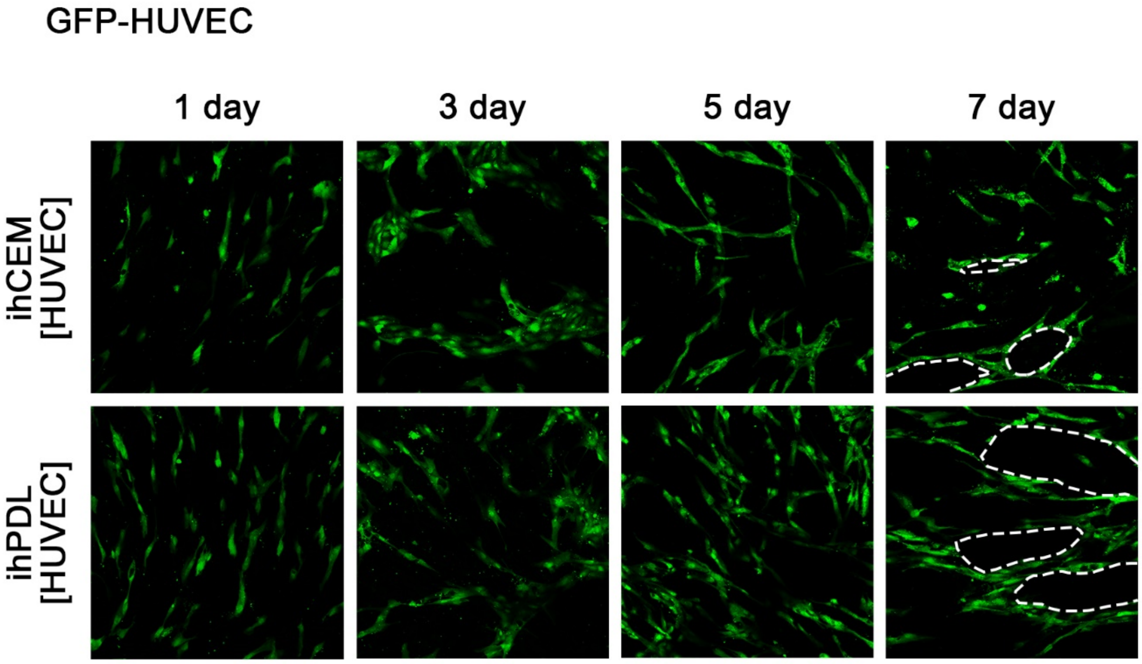

Supplementary Figure 3 | HUVEC culturing on ihCEMs and ihPDLs sheets.

HUVECs were spread onto ihCEMs and ihPDLs sheets, respectively. The photos were obtained at 1, 3, 5 and 7 days after spreading. The white dotted line indicates vasculature formation of the HUVECs at 7 days after spread on the cell sheets, respectively.

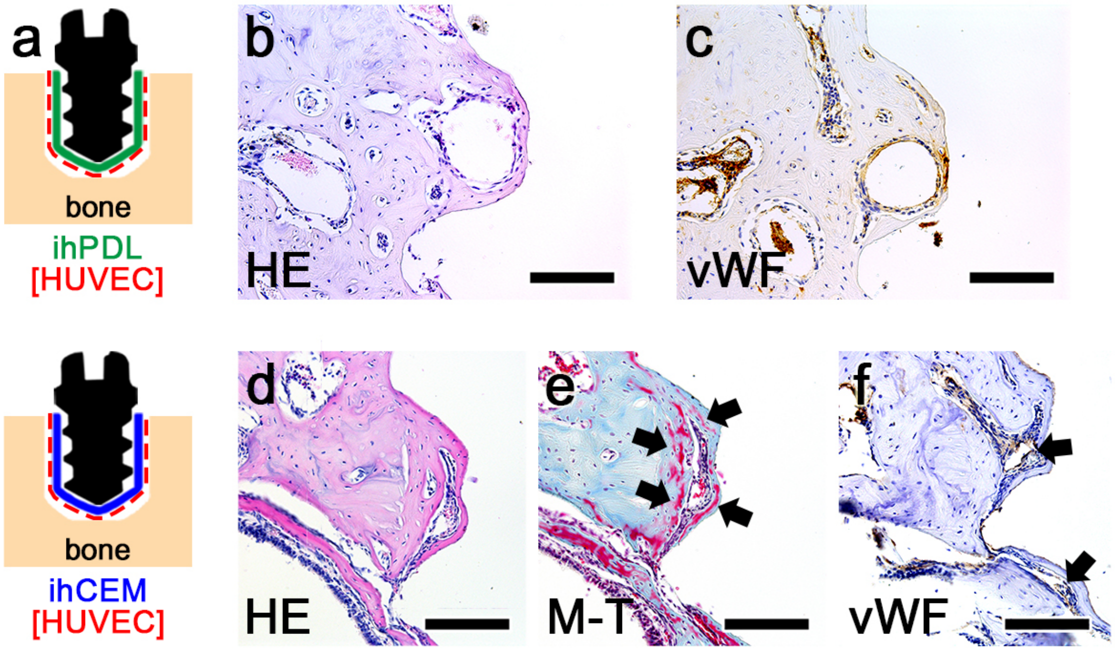

Supplementary Figure 4 | HUVEC engaged ihCEMs and ihPDLs sheets induce rapid bone growth.

(a) Schematic representation of ihPDL [HUVEC] and ihCEM [HUVEC] bio-implants. The HUVECs directly contact the alveolar bone. (b) HE-stained image of a transplanted ihPDL (HUVEC) cell sheet. Large circular vessels formed close to the fixture. The soft tissue is not shown. (c) IHC of vWF confirmed that the large circular structures were blood vessels. (d) HE-stained image of a ihCEM [HUVEC] bio-implant. The irregular shape of the blood vessels formed close to the fixture. (e) M-T-stained image of a ihCEM [HUVEC] bio-implant. Newly formed calcified tissue (arrows) was observed on both sides of the blood vessels. (f) IHC of vWF showed that the irregular-shaped structures close to the fixture surface were blood vessels (arrows). Scale bars, 100 μm.

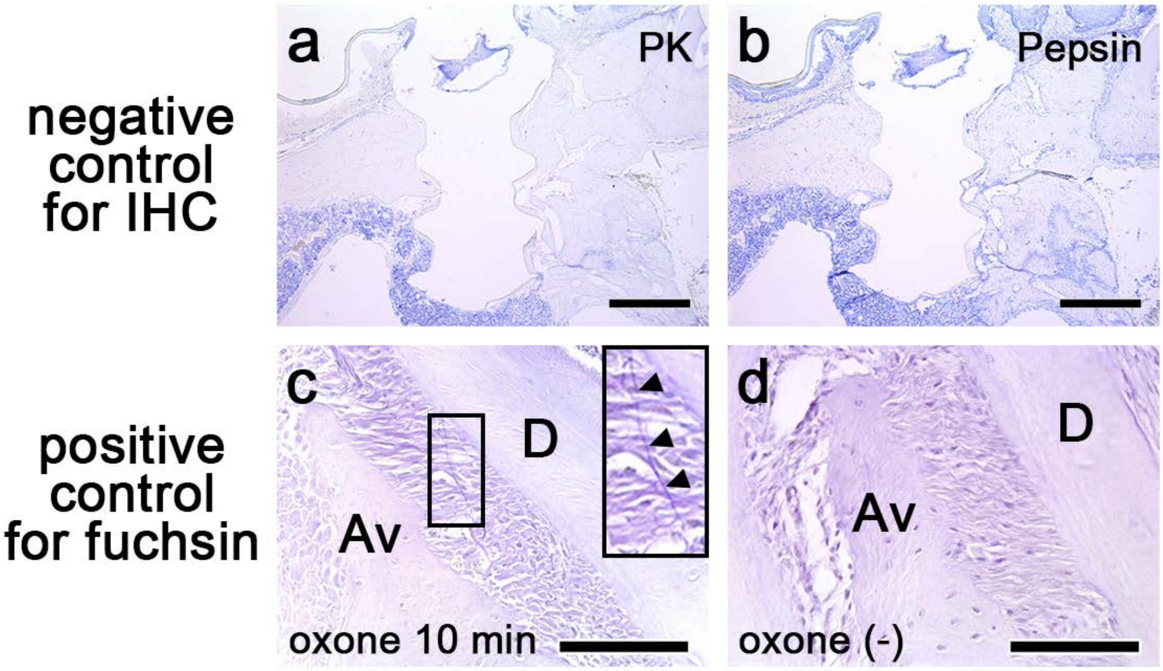

Supplementary Figure 5 | Negative control for IHC and positive control for resorcin-fuchsin staining.

(a, b) Negative control of IHC. Antigen retrieval was performed with Proteinase K and Pepsin. Primary antibody was substituted to antibody diluent (Invitrogen, USA) and goat anti rabbit IgG was applied as secondary antibody. Signal was not detected at all. (c) Positive control of Resorcin-Fuchsin staining of the mouse maxilla tooth. Oxytalan fiber was observed with oxone treatment (arrowheads). (d) Oxytalan fiber was not detected without oxone treatment. Av, Alveolar bone; D, dentin; Scale bars, a, b, 500 μm; c, d, 100 μm.

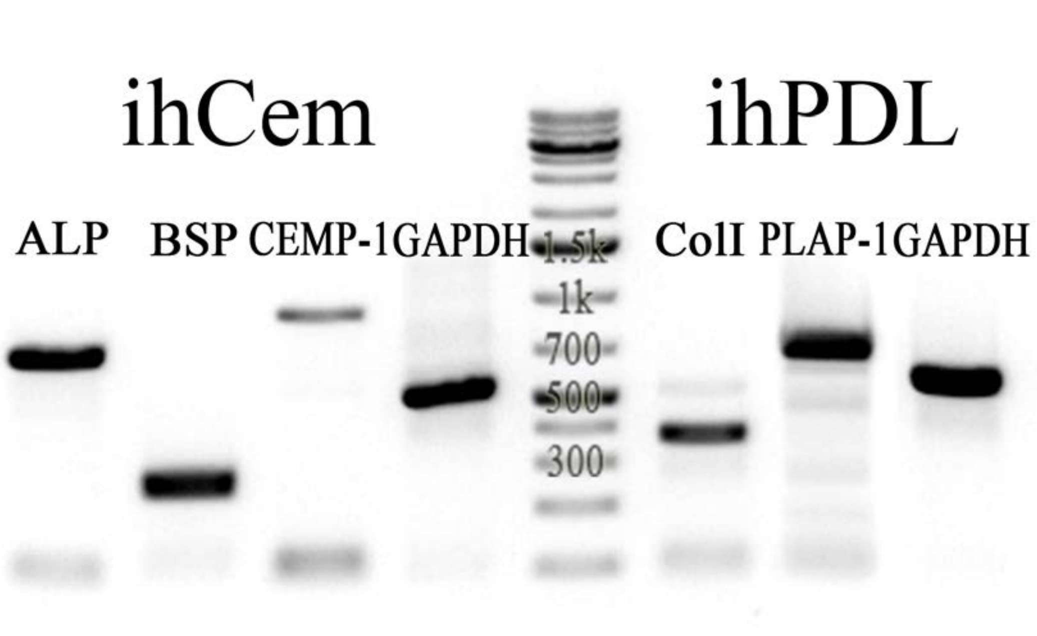

Supplementary Figure 6 | RT-PCR results of immortalized human cells (full-length gel image).

The ihCEMs expressed ALP, BSP and CEMP-1 mRNAs. The ihPDLs expressed PLAP-1 and ColI mRNAs. DNA ladder (Gene Ruler 1kb Plus DNA ladder, #SM1331, Thermo scientific, USA) was loaded in the 5th lane. All the PCR samples were loaded as twice as amount of DNA ladder. Each band of ladder contains DNA fragments as much as follow: 300bp, 700bp, 1000bp, 125ng; 500bp, 375ng; 1500bp, 400ng.
